# Sirenomelia in a preterm neonate: a rare and lethal congenital anomaly

**DOI:** 10.11604/pamj.2025.52.77.49478

**Published:** 2025-10-17

**Authors:** Fatima Zahraa Belhaj, Mohamed Sellouti

**Affiliations:** 1 Children's Hospital, Ibn Sina University Hospital Centre, Rabat, Morocco,; 2 Hôpital Militaire d'Instruction Mohammed V, Mohammed V University, Rabat, Morocco

**Keywords:** Sirenomelia, C-reactive protein, newborn, ectromelia, congenital malformation

## Image in medicine

A preterm neonate, born at 35 weeks of gestation with a birth weight of 2100 g and an Apgar score of 8/10, was delivered after an unmonitored pregnancy. Clinical examination revealed a single tapered appendage replacing the lower limbs, with absent musculature and soft tissue, highly suggestive of sirenomelia “mermaid syndrome”. External genital organs were not identifiable. Complementary investigations demonstrated severe visceral malformations: renal ultrasound revealed bilateral renal agenesis, while echocardiography showed complex congenital heart anomalies. No craniofacial dysmorphism was noted. The infant initially presented with respiratory distress requiring CPAP support. At 12 hours of life, clinical deterioration occurred with temperature instability, feeding intolerance, and increasing oxygen requirements. Laboratory investigations showed a marked rise in C-reactive protein with progressive thrombocytopenia. Despite the initiation of broad-spectrum intravenous antibiotics, the clinical course rapidly worsened. The newborn developed refractory septic shock compounded by severe renal failure with hydro-electrolyte disturbances, leading to multiorgan dysfunction and death at 48 hours of life.

**Figure 1:**
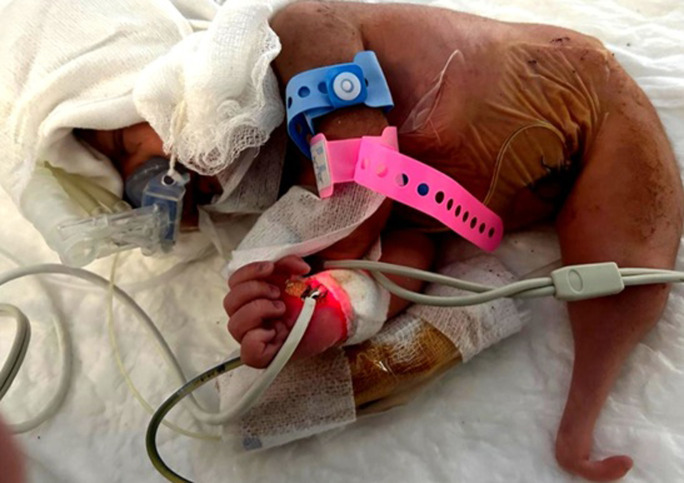
sirenomelia in a preterm neonate showing a single fused lower-limb appendage, absence of external genitalia, and associated visceral malformations

